# Bulbar Dysfunction in Idiopathic Normal Pressure Hydrocephalus: A Case Report

**DOI:** 10.7759/cureus.34579

**Published:** 2023-02-03

**Authors:** Muhammad Usama, Avneet Kaur Manjeet Singh Arora, Faraz Saleem

**Affiliations:** 1 Neurology, Sheikh Zayed Medical College/Hospital, Rahim Yar Khan, PAK; 2 Public Health and Epidemiology, University of California, Berkeley, Berkeley, USA; 3 Internal Medicine, Mahatma Gandhi Mission Medical College and Hospital, Navi Mumbai, IND; 4 Internal Medicine, Akhtar Saeed Medical and Dental College, Lahore, PAK

**Keywords:** gait ataxia, csf, vp shunt, bulbar, nph

## Abstract

Normal pressure hydrocephalus (NPH) is a rare condition characterized by pathologically enlarged ventricles and a normal cerebrospinal fluid (CSF) opening pressure measured by lumbar puncture. NPH typically presents as a triad of cognitive decline, gait disturbance, and urinary incontinence. Rarely, NPH can present with bulbar involvement, particularly with difficulty swallowing. Here, we present a case of NPH in a 75-year-old man who presented with an episode of choking and a recent onset of swallowing difficulties with a three-month history of ataxia and progressive memory loss. His CT scan revealed ventriculomegaly, which was consistent with the clinical presentation of NPH and was further confirmed by a normal opening pressure on the CSF tap. Furthermore, ventriculoperitoneal shunts showed a marked improvement in patients' dysphagia and the classical triad of NPH symptoms. Through this case report, we want to highlight that NPH can present as a difficulty in swallowing.

## Introduction

Idiopathic normal pressure hydrocephalus (NPH) is a rare neurological disorder that primarily affects the elderly. NPH is caused by ventricular enlargement, which can be caused by a CSF drainage pathway blockage, reabsorption defect, or excessive fluid production [1]. NPH can be either primary (also called "idiopathic") or secondary, depending on what caused the disease. Subarachnoid hemorrhage, head trauma, and meningitis are all secondary causes of NPH [2]. It typically manifests as the triad of urinary incontinence, gait disturbance, and cognitive impairment. Apraxia of the gait manifests first, and the triad is not always present. It was first talked about in 1965, and a ventriculoperitoneal shunt was suggested as a possible way to treat it [3]. There are few studies on the coexistence of NPH and corticobulbar tract (CBT) dysfunctions, such as stuttering and difficulty swallowing [4,5]. This case report highlights the unusual occurrence of bulbar symptoms as part of the NPH's presentation.

## Case presentation

A 75-year-old retired businessman with a recent onset of choking was brought to the Accident and Emergency (A&E) Department of the Tertiary Care Hospital. He suddenly started to choke while eating dinner because he was having trouble swallowing. He told us that he has been experiencing difficulty swallowing for the past three months, which was exacerbated particularly with liquid food. Apart from that, he has a normal appetite and has not experienced any weight loss in the recent past. Upon reviewing his medical history, he reported a three-month progression of gait instability. His wife also noticed that for the last three months, he walked with an unsteady gait, with short steps, and had difficulty trying to turn while walking. She also stated that her husband has difficulty remembering names and frequently misplaces or forgets belongings. He recently forgot his address and was having difficulty finding his way back home while roaming around the neighborhood. Later, a neighbor supported him in returning to his home.

On examination, his vital signs were stable (blood pressure: 128/77 mmHg, pulse: 79 beats per minute, temperature: 36.6°C, and oxygen saturation (SpO_2_) at room temperature: 96%). A detailed neurological examination revealed that the patient had a shuffling, short-stepping gait. His Rhomberg test was negative. His sensory and motor examinations, including cranial nerves as well as deep tendon reflexes, were all normal. There was no dysmetria or any cerebellar involvement. The review of other systems was unremarkable. His score on the Mini-Mental State Examination (MMSE) was 23.

**Figure 1:**
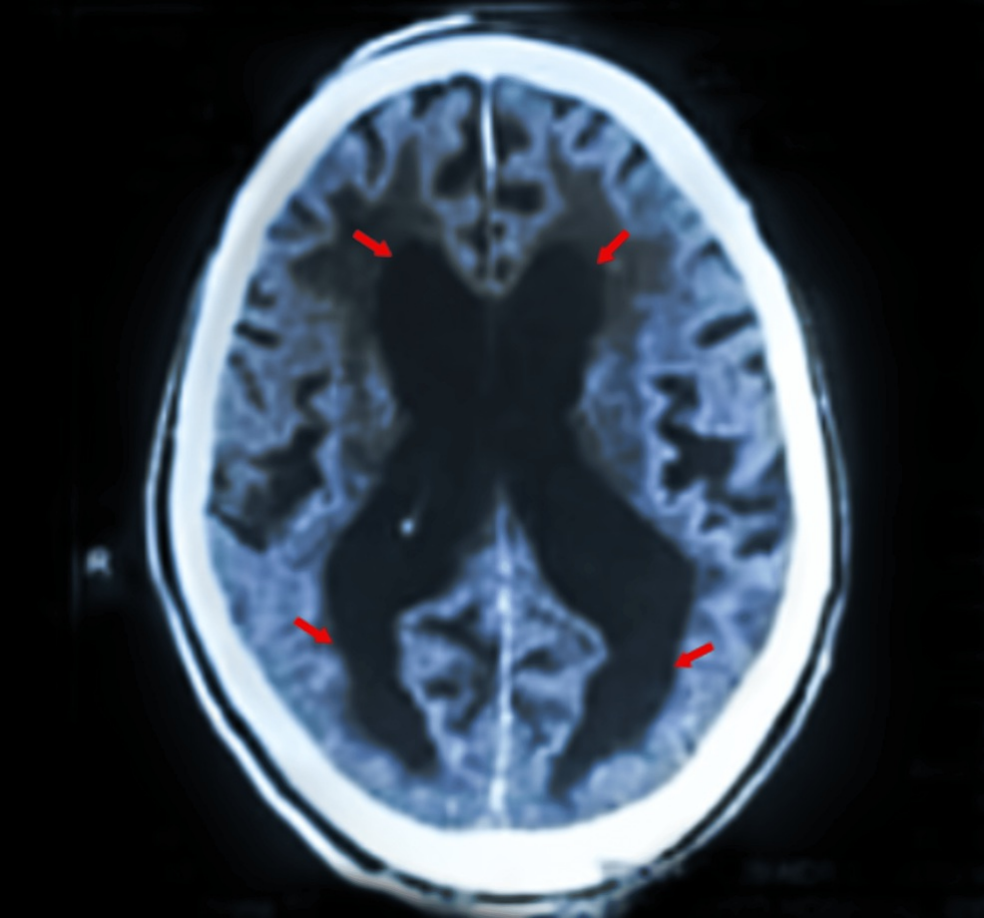
CT scan of the brain showing dilation of the lateral ventricles (arrows).

The comprehensive laboratory testing included a full blood count (FBC), urine and electrolytes (U&E), a liver function test (LFT), a C-reactive protein (CRP), thyroid function tests, serum B12 levels, and random blood sugar. All of these test results were within normal parameters. Consequently, a CT scan of the brain was performed, which revealed dilatation of the third and lateral ventricles with no hemorrhage or ischemic infarct. No mass effect or mild line shift was noted. An Evans index ratio greater than 0.31 was observed. CT scan is shown in Figure 1.

On the basis of the patient's clinical manifestations and the findings of the CT scan of the brain, normal pressure hydrocephalus was suspected. Consequently, a CSF tap was performed, revealing a normal opening pressure of 15 cmH_2_0 (normal range: 5-18 cmH_2_0). Cerebrospinal fluid (CSF) analysis revealed a normal white blood cell count of 4 WBCs per mm^3^ (normal: 0-8 leukocytes/mm^3^) and a mildly increased protein concentration of 65 mg/dL (normal: 15 to 60 mg/dL).

Following a comprehensive evaluation of the patient's clinical findings, radiological evidence, normal pressure on the CSF tap, and improvement of symptoms upon removal of 40 mL of CSF, a diagnosis of NPH was confirmed, and he was further referred to the neurosurgery team for ventriculoperitoneal (VP) shunt placement. After a successful ventriculoperitoneal shunt procedure, an immediate improvement in patient gait was noticed and later the patient was discharged home. The response to shunt placement was also assessed at three- and six-month follow-ups. The patient's gait and cognitive difficulties exhibited significant improvement. His gait improved completely, and he was able to walk and maintain his balance without assistance. In addition, his MMSE score increased from 23 to 26.

## Discussion

Normal pressure hydrocephalus is characterized by enlarged ventricles and normal cerebrospinal fluid (CSF) pressure upon lumbar puncture. The incidence of NPH varies greatly between studies, ranging from two to 20 per million people per year [1]. NPH is often characterized by a triad of walking difficulty, cognitive impairment, and urinary incontinence. NPH is a neurological disorder that is hard to diagnose because its symptoms are similar to those of dementia such as Alzheimer's, Lewy body dementia, and Parkinson's [2].

In patients with NPH, both primary (idiopathic) and secondary causes are identified. The prevalence of idiopathic NPH increases with age and is highest in persons over the age of 60. It affects both sexes with equal frequency. In a population-based study, the prevalence increased from 0.2% in those aged 70 to 79 to 6% in those over 80 [4]. Secondary NPH can affect individuals of any age group and is usually attributed to meningitis, subarachnoid hemorrhage, and trauma. In comparison to other causes of dementia in the elderly, such as Alzheimer's disease (AD), NPH is rare [2].

Although several potential mechanisms of idiopathic NPH have been proposed, no apparent cause has been identified. Several studies have found a strong correlation between decreased CSF absorption and a higher incidence of idiopathic NPH. In up to 50% of cases, arachnoid thickening is one of the autopsy findings in some patients with NPH [4]. In one of the studies, individuals with NPH have larger head diameters than normal controls, suggesting that some patients with NPH may well have congenital hydrocephalus that manifests later in life [4]. Moreover, individuals with idiopathic NPH have a higher prevalence of hypertension, coronary artery disease, peripheral arterial disease, and other vascular risk factors compared to age-matched controls, indicating that vascular disease is anticipated to occur more frequently in these patients.

Very little is known about the presentation of NPH as bulbar symptoms, such as speech difficulties and swallowing problems. Adams noted in 1975 that a few of the patients who had the classic triad of NPH also had speech difficulties ranging from difficulty articulating or whispering until it eventually became nonexistent [5]. In one prospective study, Chankaew et al. reported that 43 out of 53 patients with normal pressure hydrocephalus had swallowing problems prior to shunt placement [6]. They also experienced aspiration pneumonia secondary to choking. A substantial relationship existed between swallowing impairment, speech difficulty, and the NPH classical triad [6]. One study stated that the severity of bulbar symptoms was directly associated with the increasing severity of the NPH triad [6]. Eleftheriou et al. also documented bulbar dysfunction as one of the manifestations of NPH [6,7]. 

There are two proposed pathological reasons for bulbar dysfunction in NPH. The first one is the hypoperfusion surrounding the Sylvian fissure region, which is the site of the laryngeal motor cortex and swallowing. The perisylvian cortical hypoperfusion can be explained by Sylvian cistern dilatation, which is a typical observation in idiopathic NPH. The second cause is periventricular white matter damage (PVWM). This region has both corticospinal tract (CST) and CBT adjacent to the lateral ventricle. Hence, dilation of the ventricle stretches these fibers and has a direct impact on CBT. CBT can be skewed by either larger ventricles or decreased cerebral blood flow (CBF) [6]. Momjian et al. discovered that in NPH, a decrease in white matter regional CBF was correlated with the triad of NPH [8]. As a result, greater perfusion in these locations following shunt implantation has resulted in improved bulbar dysfunction [6]. Mathew et al. reported that NPH could present as stuttering and dysarthria [2]. With the help of a VP shunt, CSF drainage helped relieve these classic NPH triad symptoms as well as bulbar symptoms like stuttering [2].

NPH is commonly identified and treated based on clinical symptoms, CSF dynamics, and brain imaging. Given the invasive nature of the treatment for NPH and the high failure rate, it is generally recommended that an additional test be performed to determine the likelihood that the patient will respond to surgery [4]. A trial lumbar drain uses a temporary catheter to let CSF drain from the lumbar CSF space at a rate of 5-10 mL/hour. During a two- to seven-day hospital observation period, clinicians, patients, and family members assess the clinical response.

When doing a lumbar puncture or a lumbar drain trial, it makes sense to do routine diagnostic tests on the CSF, such as a cell count, a protein test, and a glucose test. In the majority of cases of idiopathic NPH, these test results are expected to be normal, although a mild elevation of protein in isolation is a common nonspecific finding [4]. A ventricular shunt implant is used to treat NPH. The majority of shunts transfer CSF from a catheter in the lateral ventricle into the abdomen (ventriculoperitoneal shunt). A study was done to compare the preoperative and postoperative effects of bulbar dysfunction after shunt placement in patients with normal pressure hydrocephalus. Six months after a ventricular shunt was placed, 86% of patients reported considerable improvement in their swallowing and speech difficulties [6]. According to Japan's limited data, lumboperitoneal shunting may be a viable alternative to effective cranial surgery [4].

## Conclusions

This case report highlights the unusual occurrence of bulbar symptoms as part of the NPH's presentation. As a clinician, one should be aware that NPH can manifest itself in a variety of ways other than the classic triad of ataxia, memory impairment, and urinary incontinence. It must be distinguished from dementia, stroke, and other brain pathologies that frequently appear with bulbar involvement in this age group. As NPH is reversible and has a good prognosis, prompt identification and treatment are critical for patient survival.
